# The Roles of Diacylglycerol Kinase α in Cancer Cell Proliferation and Apoptosis

**DOI:** 10.3390/cancers13205190

**Published:** 2021-10-16

**Authors:** Fumio Sakane, Fumi Hoshino, Masayuki Ebina, Hiromichi Sakai, Daisuke Takahashi

**Affiliations:** 1 Department of Chemistry, Graduate School of Science, Chiba University, Chiba 263-8522, Japan; f.hoshino@chiba-u.jp (F.H.); mebina@chiba-u.jp (M.E.); 2 Department of Biosignaling and Radioisotope Experiment, Interdisciplinary Center for Science Research, Organization for Research and Academic Information, Shimane University, Izumo 693-8501, Japan; hisakai@med.shimane-u.ac.jp; 3 Department of Pharmaceutical Health Care and Sciences, Kyushu University, Fukuoka 812-8582, Japan; dtakahashi@phar.kyushu-u.ac.jp

**Keywords:** diacylglycerol kinase, calcium, tyrosine phosphorylation, protein kinase C, nuclear factor-κB, ERK, mammalian target of rapamycin, Ras-guanyl nucleotide-releasing protein, programmed cell death-1, anticancer immunity

## Abstract

**Simple Summary:**

Diacylglycerol (DG) kinase (DGK) phosphorylates DG to generate phosphatidic acid (PA). DGKα is highly expressed in several refractory cancer cells, including melanoma, hepatocellular carcinoma, and glioblastoma cells, attenuates apoptosis, and promotes proliferation. In cancer cells, PA produced by DGKα plays an important role in proliferation/antiapoptosis. In addition to cancer cells, DGKα is highly abundant in T cells and induces a nonresponsive state (anergy), representing the main mechanism by which advanced cancers avoid immune action. In T cells, DGKα induces anergy through DG consumption. Therefore, a DGKα-specific inhibitor is expected to be a dual effective anticancer treatment that inhibits cancer cell proliferation and simultaneously activates T cell function. Moreover, the inhibition of DGKα synergistically enhances the anticancer effects of programmed cell death-1/programmed cell death ligand 1 blockade. Taken together, DGKα inhibition provides a promising new treatment strategy for refractory cancers.

**Abstract:**

Diacylglycerol (DG) kinase (DGK) phosphorylates DG to generate phosphatidic acid (PA). The α isozyme is activated by Ca^2+^ through its EF-hand motifs and tyrosine phosphorylation. DGKα is highly expressed in several refractory cancer cells including melanoma, hepatocellular carcinoma, and glioblastoma cells. In melanoma cells, DGKα is an antiapoptotic factor that activates nuclear factor-κB (NF-κB) through the atypical protein kinase C (PKC) ζ-mediated phosphorylation of NF-κB. DGKα acts as an enhancer of proliferative activity through the Raf–MEK–ERK pathway and consequently exacerbates hepatocellular carcinoma progression. In glioblastoma and melanoma cells, DGKα attenuates apoptosis by enhancing the phosphodiesterase (PDE)-4A1–mammalian target of the rapamycin pathway. As PA activates PKCζ, Raf, and PDE, it is likely that PA generated by DGKα plays an important role in the proliferation/antiapoptosis of cancer cells. In addition to cancer cells, DGKα is highly abundant in T cells and induces a nonresponsive state (anergy), which represents the main mechanism by which advanced cancers escape immune action. In T cells, DGKα attenuates the activity of Ras-guanyl nucleotide-releasing protein, which is activated by DG and avoids anergy through DG consumption. Therefore, a DGKα-specific inhibitor is expected to be a dual effective anticancer treatment that inhibits cancer cell proliferation and simultaneously enhances T cell functions. Moreover, the inhibition of DGKα synergistically enhances the anticancer effects of programmed cell death-1/programmed cell death ligand 1 blockade. Taken together, DGKα inhibition provides a promising new treatment strategy for refractory cancers.

## 1. Introduction

**Figure 1:**
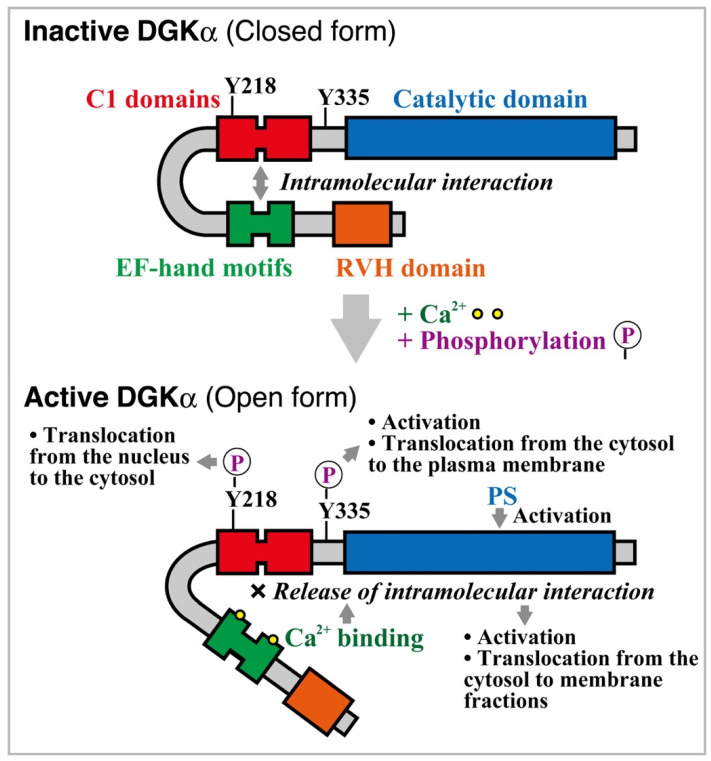
Structure and activation mechanisms of DGKα. DGKα has a recoverin homology (RVH) domain, two Ca^2+^-binding EF-hand motifs, two C1 domains, and a catalytic domain. Ca^2+^-dependent detachment of the intramolecular interaction between the EF-hand motifs and the C1 domains is the crucial event that controls DGKα activity and subcellular localization (translocation from the cytosol to membrane fractions). Phosphatidylserine (PS) binds to the catalytic domain and enhances activity. Phosphorylation at Tyr-335 induces translocation from the cytosol to the plasma membrane and activation of DGKα. Phosphorylation at Tyr-218 induces translocation from the nucleus to the cytosol.

Diacylglycerol kinase (DGK) phosphorylates diacylglycerol (DG, 1,2-diacyl-*sn*-glycerol) to generate phosphatidic acid (PA, 1,2-diacyl-*sn*-glycerol-3-phosphate) [1,2,3,4,5]. Mammalian DGK consists of ten isozymes, which are divided into five groups (type I (α, β, and γ), II (δ, η, and κ), III (ε), IV (ζ and ι) and V (θ)) according to their structural features. The α isozyme of DGK (one of the type I isozymes) was first cDNA-cloned approximately 30 years ago [6,7]. This isozyme characteristically has two tandem EF-hand motifs at its N terminus (Figure 1). DGKα is abundantly expressed in several cancer cell lines including hepatocellular carcinoma, melanoma, glioblastoma, colon adenocarcinoma, and breast adenocarcinoma cells, enhancing their progression and proliferation, and attenuating apoptosis [8,9,10,11]. Therefore, the inhibition of DGKα activity is expected to suppress the progression and proliferation of these cancers. In contrast, DGKα, which is also highly expressed in T lymphocytes, facilitates the nonresponsive state known as T cell clonal anergy [12,13]. Anergy induction in T lymphocytes is the primary mechanism by which progressive tumors evade immunity. Therefore, if a DGKα-specific inhibitor is developed, it would reversely and simultaneously inhibit tumor cell proliferation and enhance T cell function and consequently can be a dual effective drug. In this article, we review the properties of DGKα and its roles in cancer cell proliferation and apoptosis, and anticancer immunity.

## 2. Structure, Enzymatic Properties, and Activation Mechanisms of DGKα

### 2.1. Structure

DGKα is a member of type I DGK [1,2,3,4,5]. This isozyme contains, from its N terminus, a recoverin homology (RVH) domain [14], two tandem EF-hand motifs, two tandem C1 domains, and a catalytic domain [15] (Figure 1). Indeed, two Ca^2+^ ions bind to two EF-hand motifs (one each) in DGKα and strongly activate it [6,16]. The apparent dissociation constant *K*_D_ and ED_50_ value of DGKα for Ca^2+^ are approximately 0.3 μM [16,17]. In contrast to DGKα, DGKβ and DGKγ, which also have two tandem EF-hand motifs, are apparently active independent of Ca^2+^ [17]. The apparent *K*_D_ values of DGKβ and DGKγ for Ca^2+^ are less than 0.01 μM [17]. Therefore, although DGKβ and DGKγ have a stronger affinity for Ca^2+^ than DGKα, they failed to show Ca^2+^-dependent activation, suggesting that pretreatment with a Ca^2+^ chelator, ethylene glycol tetraacetic acid (EGTA), cannot effectively release Ca^2+^ from these isozymes. The concentrations of Ca^2+^ in resting and stimulated cells are 0.05–0.1 nM and 1–10 μM, respectively [18,19]. Thus, it is likely that EF-hand motifs in DGKα associate with Ca^2+^, and the isozyme is activated by Ca^2+^ only after cell stimulation. In addition to activation, Ca^2+^ induces translocation of DGKα to membrane fractions [16].

The C1 domains are similar to those in protein kinase C (PKC). The domains in conventional and novel PKC isoforms bind to phorbol ester and DG [20,21]. However, the C1 domains in DGKα showed no phorbol ester-binding activity, and the catalytic domain alone, lacking the C1 domains, exhibited catalytic (DG recognition and phosphorylation) activity [15], indicating that the domains do not act as the catalytic domain.

Site-directed mutagenesis analyses showed that the N- and C-terminal regions of the catalytic domain bind to ATP and DG, respectively [22]. Miller et al. studied the 3D structure of *Staphylococcus aureus* DGK (DgkB), which is structurally similar to the catalytic domains of mammalian DGKs and its key active site residues [23]. However, 3D structures of full-length mammalian DGK isozymes have not yet been revealed. Nonetheless, the key active site residues and the components of the Asp–water–Mg^2+^ network are conserved in the catalytic cores of mammalian DGKs. There are remarkable similarities among ceramide kinase, sphingosine kinase, and DGK catalytic domains [24]. Therefore, it is possible that these enzymes utilize the same mechanism and have 3D structures similar to that of DgkB.

### 2.2. Enzymological Properties

DGKα generates PA using ATP and DG as substrates. The *K*_m_ values for ATP and DG are 0.10–0.24 mM and 1.0–3.4 mol%, respectively, which are close to averages of DGK isozymes measured thus far [15,16,25,26].

Interestingly, in addition to DGK activity, DGKα has 2-monoacylglycerol (MG) kinase (MGK) activity (approximately 12% of DGK activity) but not 1-MGK activity [27]. Type I (α, β, and γ), II (δ, η, and κ) and III (ε) DGK isozymes also have 2-MGK activity (12–19% of DGK activity). However, type IV DGK isozymes (ζ and ι) do not show 1-MGK or 2-MGK activity (less than 1% of DGK activity). Alternatively, DGKθ (type V) has 1-MGK activity (6% of DGK activity) but not 2-MGK activity. DGK isozymes with 1-MGK or 2-MGK activity may produce lysoPA, utilizing 1-MG or 2-MG in cells. However, the physiological significance of their 1-MGK and 2-MGK activities is still unknown. Notably, intracellular lysoPA is involved in cancer cell migration [28].

We recently found, using liquid chromatography (LC)–tandem mass spectrometry (MS/MS), that the production of palmitic acid (16:0)- and/or palmitoleic acid (16:1)-containing PA species, such as 14:1/16:1-, 14:0/16:1-, 14:0/16:0-, 16:1/16:2-, 16:1/16:1-, 16:0/16:1-, 16:0/16:0-, 16:0/18:1- and 16:0/18:0-PA, were inhibited by CU-3, a DGKα-selective inhibitor [29] (see Section 5.1), in starved Jurkat T cells [30]. Moreover, LC–MS/MS revealed that the production of 16:0-containing PA species, such as 16:0/16:0- and 16:0/18:0-PA, was attenuated by CU-3 [29] in AKI melanoma cells under starved conditions [31]. These results strongly suggest that DGKα phosphorylates different DG species in cancer cells and T cells. DGKα does not exhibit DG species selectivity in vitro [25]. It is possible that DG supply enzymes, which provide distinct DG species upstream of DGKα, are different from each other in cancer and T cells. However, further studies are needed to identify the upstream enzymes.

DGK activity (conversion of DG to PA) is essential for phosphatidylinositol (PI) turnover (the PI 4,5-bisphosphate pathway) as the initial step for PI resynthesis [32,33]. As PI turnover exclusively generates 1-stearoyl-2-arachidonoyl-DG (18:0/20:4-DG), it has been generally considered that all DGK isozymes phosphorylate 18:0/20:4-DG species. However, our group recently revealed that DGK isoforms (DGKα, δ, η, and ζ) except for DGKε, phosphorylate a variety of DG molecular species, which are not coming from the PI 4,5-bisphosphate pathway [4,30,34,35,36,37,38,39,40].

Recently, Bozelli et al. reported that membrane morphology (membrane curvature) affects the substrate selectivity of DGKα [41]. On substantial membrane morphological changes, DGKα exhibits higher substrate acyl chain specificity for 16:0-containing DG molecular species, which are actually preferred by DGKα as substrates in cells [30,31,42]. It is possible that DGKα (and other DGK isozymes) metabolize specific molecular species of DG and, consequently, generate specific molecular species of PA in a membrane morphology-dependent manner.

### 2.3. Regulation of Activity and Subcellular Localization 

Several studies have revealed calcium-dependent activation mechanisms of DGKα [43,44,45] (Figure 1). The N-terminal region containing the EF-hand motifs and RVH domain masks the catalytic domain and maintains the enzyme in an inactive state. Ca^2+^ induces the release of the intramolecular association between the C1 domains and the EF-hand motifs of DGKα (Figure 1). Therefore, dissociation between the EF-hand motifs and the C1 domains is the key event that activates DGKα (Figure 1). Experiments with 2-*p*-toluidinylnaphthalene 6-sulfonate, a probe for hydrophobic regions of proteins, showed that the binding of Ca^2+^ masked a hydrophobic region of DGKα EF-hand motifs [17]. We recently succeeded in the first crystal structure of Ca^2+^-bound DGKα EF-hand motifs and analyzed the structural changes on binding to Ca^2+^ [46]. The EF-hand motifs of DGKα adopt a canonical EF-hand fold but unpredictably possess a ligand mimic helix (an additional α-helix), which is packed into the hydrophobic core [46]. Conformational changes may contribute to the dissociation of intramolecular interactions between the EF-hand motifs and the C1 domains in DGKα and its activation (Figure 1).

It has been repeatedly reported that, similar to PKC [47,48], an acidic phospholipid, phosphatidylserine (PS), enhances DGKα activity [15,16,25,26]. Intriguingly, Ca^2+^ and PS stimulate DGKα enzyme activity via distinct mechanisms [22]. Although Ca^2+^ interacts with EF-hand motifs as described above, PS binds to the catalytic domain (Figure 1). Unlike DGKα, PKC interacts with PS through its C2 domain but not the catalytic domain [47,48].

In addition to PS, phosphoinositide 3-kinase lipid products, such as PI 3,4-bisphosphate and PI 3,4,5-trisphosphate, activate DGKα [49]. Interestingly, this activation occurs in a calcium-independent manner. In addition to acidic phospholipids, sphingosine, which is a basic lipid and forms a primary part of sphingolipids including sphingomyelin, activates DGKα in vitro and in cells [50,51,52].

Phosphorylation at Tyr-335 in DGKα (Tyr-335 in human DGKα (https://www.uniprot.org/uniprot/P23743 (accessed on 25 September 2021)); Tyr-334 in pig DGKα (https://www.uniprot.org/uniprot/P20192 (accessed on 25 September 2021)); Tyr-336 in mouse DGKα (https://www.uniprot.org/uniprot/O88673 (accessed on 25 September 2021)) is also involved in its activation and subcellular localization (Figure 1). In T cells, DGKα translocates from the cytosol to the plasma membrane in response to T cell receptor stimulation [53]. Tyr-335 phosphorylation induces the plasma membrane translocation of the enzyme in T cells [54]. In addition to T cell receptor, D-α-tocopherol (vitamin E) induces Src-dependent phosphorylation at Tyr-335 in DGKα, and consequently, the phosphorylation enhances translocation from the cytoplasm to the plasma membrane and activation of the enzyme in DDT1-MF2 cells (a smooth muscle cell line) [55] (Figure 1). Epigallocatechin gallate (a green tea polyphenol) also induces the Src-dependent tyrosine phosphorylation of DGKα and consequently causes its translocation and activation in DDT1-MF2 cells [56]. The effects of D-α-tocopherol and epigallocatechin gallate occur through a 67 kDa laminin receptor [57].

Tyr-218 in DGKα is phosphorylated by c-Abl [58] (Figure 1). Tyr-218 phosphorylation regulates serum-induced nuclear export of DGKα.

As described above, various signal transduction routes intricately regulate the activity and subcellular localization of DGKα activity.

## 3. Regulation of Cancer Cell Proliferation and Apoptosis by DGKα

**Figure 2:**
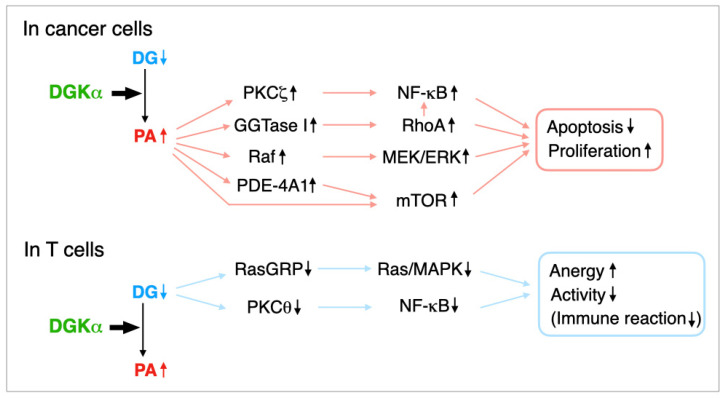
Proposed pathways utilized by DGKα in cancer cells and T lymphocytes. In cancer cells, DGKα acts as an antiapoptosis/proproliferation factor via the PKCζ–NF-κB, GGTase I–RhoA(–NF-κB), Raf–MEK–ERK and PDE-4A1–mTOR pathways. As PKCζ, Raf, PDE-4A1, and mTOR are PA-binding proteins and PA activates them, it is likely that PA generated by DGKα mediates the antiapoptosis and proproliferation effects of the enzyme. In T cells, DGKα acts as an immune checkpoint and promotes the nonresponsive state known as clonal anergy through the inactivation of Ras-guanyl nucleotide-releasing protein (RasGRP), which is activated by DG and activates the Ras–MAPK pathway. In addition, DGKα attenuates the PKCθ–NF-κB pathway, which is also important for preventing T cell anergy.

We demonstrated that DGKα is expressed in several human melanoma cell lines including AKI but not in noncancerous normal human epidermal melanocytes and that the isozyme suppresses tumor necrosis factor-α-induced apoptosis of AKI melanoma cells through the activation of nuclear factor-κB (NF-κB) [11], which is an antiapoptotic factor (Figure 2). DGKα activates NF-κB through PKCζ-dependent phosphorylation at Ser-311 of the p65/RelA subunit of NF-κB in AKI melanoma cells [59] (Figure 2).

In addition to melanoma cells, Takeishi et al. demonstrated that DGKα expression is correlated with hepatocellular carcinoma (HCC) progression [9]. Moreover, DGKα enhances HCC proliferation by activating extracellular signal-regulated kinase (ERK) [9], which is downstream of Ras–Raf–mitogen-activated protein kinase (MAPK)/ERK kinase (MEK) (Figure 2).

Dominguez et al. reported that DGKα inhibition using R59022 and R59949, which are commercially available semi-DGKα-selective inhibitors (see Section 5.1), and silencing induced apoptosis of several cancer cell lines including brain glioblastoma and melanoma cells [8] (Figure 2). In addition, they demonstrated that the apoptotic effects are mediated by the phosphodiesterase (PDE)-4A1–cAMP–mammalian target of rapamycin (mTOR) pathway [8] (Figure 2). In this case, DGKα inhibition/silencing decreased mTOR expression [8].

PA produced by DGKα activates the geranylgeranyltransferase (GGTase) I–RhoA and consequently GGTase I–RhoA–NF-κB pathways in glioblastoma cells [60] (Figure 2). GGTase I activated by PA geranylgeranylates RhoA and induces its membrane localization and activation. Geranylgeranylated RhoA and NF-κB activated by RhoA prevent cell death. Therefore, DGKα confers the mesenchymal phenotype, which is characterized by aggressiveness and treatment resistance, to glioblastoma cells.

The proliferation of colon and breast cancer cell lines was markedly suppressed by DGKα-siRNA and R59949 [10]. Moreover, DGKα interacts with Src and promotes Src activation [10]. Interestingly, Perez et al. recently demonstrated that the Src unique and SH3 domains bind to acidic phospholipids, including PA, suggesting the presence of a previously unrecognized additional regulatory mechanism of c-Src [61]. Therefore, in addition to conventional pathways, PA may activate Src in cancer cells and promote cancer cell proliferation.

In addition to the effects on apoptosis and proliferation, DGKα is reportedly essential for matrix invasion of breast carcinoma cells through the atypical PKC–β1 integrin signaling pathway [62].

DGKα is abundant in the nuclei of human erythroleukemia K562 cells and promotes cell proliferation by control of the retinoblastoma protein (pRb) phosphorylation and cell cycle progression through the G1/S checkpoint [63]. R59022, R59949, and DGKα knockdown significantly reduced K562 cell proliferation [63].

It is important to know how DGKα expression is regulated in cancer cells. Kefas et al. reported that microRNA-297 (miR-297) strongly reduced DGKα protein expression [64]. Moreover, miR-297 decreased glioblastoma cell survival, invasiveness, and tumorigenicity [64].

Taken together, inhibition of DGKα suppresses cancer cell proliferation, enhances cancer cell apoptosis, and attenuates cancer cell invasion. As PKCζ [65], GGTase I–RhoA(–NF-κB) [60], C-Raf (Raf-1) [66,67,68] and PDE-4A1 [69,70] are PA-binding proteins, and PA augments their activities, it is likely that PA produced by DGKα mediates the antiapoptosis and proproliferation effects of DGKα (Figure 2). PA interacts with and activates mTOR in addition to PDE-4A1 [71]. Therefore, PA may synergistically enhance the PDE-4A1–mTOR pathway. It is possible that PA simultaneously utilizes these three pathways, or, alternatively, may use a distinct pathway in different cancer cells.

## 4. Regulation of T Cell Receptor Signaling by DGKα

In addition to cancer cells, DGKα is abundantly expressed in T cells and the thymus [6,72]. DGKα serves as an immune checkpoint and induces T cell clonal anergy (the nonresponsive state) [12,13]. Anergy induction in T lymphocytes by progressive tumors is the primary machinery employed to escape immunological strike [73]. Particularly, DGKα reduces the antitumor immune reaction of tumor-infiltrating CD8^+^ T cells [74]. Therefore, the inhibition of DGKα activity is widely considered to enhance T cell proliferation/functions, which provide boosted immune surveillance and cancer immunity [75,76,77,78,79,80].

In addition to conventional and novel PKCs, DG activates Ras-guanyl nucleotide-releasing protein (RasGRP) [81], which drives Ras and the MAPK pathway (ERK and c-Jun N-terminal kinase (JNK), along with promoting the transcriptional activity of nuclear factor of activated T cells and the expression of interleukin-2 and CD25 (Figure 2). Indeed, DGKα reduces RasGRP1 activity through consumption of DG and, consequently, induces T cell anergy [13,53,82].

PKCθ (novel PKC), which is activated by DG [20,21], is expressed in T lymphocytes in a relatively selective manner and DGKα attenuates the PKCθ–NF-κB pathway, which is also important for preventing T cell anergy [83]. In this case, PKCθ activates IκB kinase β, leading to NF-κB activation [83].

What is the difference in DGKα functions between T lymphocytes and cancer cells? DGKα consumes DG and produces PA (Figure 2). It is likely that in T cells, DG, not PA, primarily attenuates anergy and stimulates functions and that, in contrast, PA, not DG, mainly promotes cell proliferation and suppresses apoptosis in cancer cells (Figure 2). Thus, it is speculated that DGKα-dependent consumption of DG and the production of PA dominantly affect T cells and cancer cells. As described above (see Section 2.2), DGKα consumes different DG molecular species in AKI melanoma cells (16:0/16:0- and 16:0/18:0-PA) [31] and Jurkat T cells (16:0- and/or 16:1-containing DG species) under starved conditions [30]. These results suggest that DG/PA molecular species having different fatty acid moieties in T cells and cancer cells may contribute to distinct functions of DGKα in these cells.

It is also important to know how DGKα expression is regulated in T cells. We analyzed the 5′-upstream region (3.4 kb) of the DGKα gene and revealed that the transcription of DGKα is differently regulated in human Jurkat T cells and human hepatocellular carcinoma HepG2 cells [84]. Moreover, Zheng et al. demonstrated that transcriptional regulator early growth response gene 2 (Egr2) up-regulates DGKα expression and is required for T cell anergy [85]. Therefore, it is likely that DGKα expression is differently regulated in immune and cancer cells.

## 5. DGKα Inhibitors Simultaneously Attenuate Cancer Cell Proliferation and Activate T Cell Function

### 5.1. DGKα Inhibitors Simultaneously Attenuate Cancer Cell Proliferation and Activate T Cell Function

**Figure 3:**
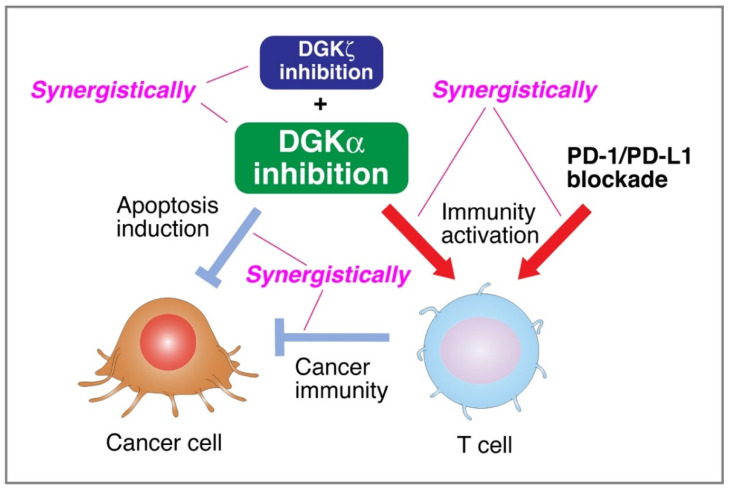
DGKα-selective inhibitors would be dual effective anticancer medicines that inhibit cancer cell proliferation and simultaneously activate T cell function. It is likely that apoptosis induction of cancer cells (direct effect) and cancer immunity (indirect effect) induced by DGKα inhibition synergistically cause damage to cancer cells. Moreover, the inhibition of DGKζ can synergistically enhance the effects of DGKα inhibition. Furthermore, the cooperative effect observed after PD-1/PD-L1 blockade and DGKα inhibition offers a promising strategy to improve the efficacy of immunotherapy in the treatment of cancer.

DGKα-selective inhibitors are expected to be dual effective drugs (i.e., ideal cancer therapy medicines) because, as described above (Section 3 and Section 4), they simultaneously suppress cancer cell proliferation and boost immune reactions, including anticancer immunity [79] (Figure 3). Several inhibitors of DGK isozymes, including DGKα, were identified as follows:

There are two DGK inhibitors, 6-{2-{4-[(4-fluorophenyl)phenylmethylene]-1-piperidinyl}ethyl}-7-methyl-5H-thiazolo-(3,2-a)pyrimidin-5-one (R59022) [86] and 3-{2-(4-[bis-(4-fluorophenyl)methylene]-1-piperidinyl)ethyl}-2,3-dihydro-2-thioxo-4(1H)quinazolinone (R59949) [87], which are commercially available. It was reported that they are selective for a calcium-dependent DGK isoform [88]. However, our comprehensive analyses of their effects on all DGK isoforms revealed that R59022 inhibited DGKα (type I), ε (type III) and θ (type V) and that R59949 inhibited DGKα (type I), γ (type I), δ (type II), and κ (type II) [89]. Therefore, these inhibitors semiselectively inhibit DGK isozymes. The half-maximal inhibitory concentration (IC_50_) values of R59022 and R59949 were 25 μM and 18 μM, respectively [89]. These values are considerably higher than those of newly found DGKα-selective inhibitors (see below).

We recently reported that high-throughput screening (HTS) of the core library (Drug Discovery Initiative, the University of Tokyo) that consists of 9600 compounds identified CU-3, 5-((2E)-3-(2-furyl)prop-2-enylidene)-3-[(phenylsulfonyl)amino]-2-thioxo-1,3-thiazolidin-4-one, as a DGKα-selective inhibitor with an IC_50_ value of 0.6 μM [29]. The IC_50_ values of CU-3 against nine other isozymes (β–κ) were 12- to 60-fold higher than those of DGKα [29]. As expected, CU-3 induced both apoptosis of several cancer-derived cell lines and T-cell activation [29,31] (Figure 3). Apoptosis induction of cancer cells (direct effect) and cancer immunity (indirect effect) induced by DGKα inhibition synergistically cause damage to cancer cells (Figure 3).

We obtained compound A, which was modified from a small molecule that was newly identified by HTS of chemical compound libraries in Ono Pharmaceutical Co., Ltd., and found that compound A also selectively inhibits type I DGK isozymes (DGKα, DGKβ, and DGKγ) but not type II–V isozymes, with IC_50_ values of 0.04, 0.02 and 0.01 μM, respectively [31]. The IC_50_ values for the other seven DGK isozymes (type II–V) were greater than 10 μM. Compound A also induced apoptosis of several cancer-derived cells and simultaneously activated T cells [31]. The DGKα-selective inhibitor DGKAI, which was obtained along with compound A (see above), also selectively inhibited DGKα, DGKβ and DGKγ with IC_50_ values of 0.01, 0.01, and <0.01 μM, respectively [90]. However, the IC_50_ values for the other seven DGK isozymes were greater than 10 μM. DGKAI was suggested to have dual effects on HCC proliferation (inhibition) and T cell immune response (activation) in vivo [90] (Figure 3). Unfortunately, structural information on these compounds is not currently available.

Other DGKα-selective inhibitors have been identified. Ritanserin, 6-[2-[4-[bis(4-fluorophenyl)methylidene]piperidin-1-yl]ethyl]-7-methyl-[1,3]thiazolo[2,3-b]pyrimidin-5-one, is a well-known serotonin receptor (5-HT_2A_/5-HT_2c_) antagonist with high structural similarity with both R59022 and R59949. Interestingly, Boroda et al. showed that ritanserin selectively inhibits DGKα (IC_50_ = 15.1 μM)) [60,91]. Moreover, Velnati et al. performed an in silico approach based on chemical homology with R59022 and R59949 and, as a result, found ritanserin and AMB639752 (1-(2,5-dimetil-1H-indol-3-yl)-2-(4-(furan-2-carbonil)piperazin-1-yl)etan-1-one), which selectively inhibit DGKα [92]. These compounds are also expected to be able to become ideal anticancer medicines.

### 5.2. Synergistic Effect of PD-1/PD-L1 Blockade

Immune checkpoint inhibitors, including anti-programmed cell death-1 (PD-1) and anti-PD-1 ligand (PD-L1) antibodies, have exhibited remarkable efficacy in some advanced cancers [93,94]. However, the clinical response rate to anti-PD-1/PD-L1 antibodies is still 10–40% in different advanced cancers [95,96], restricting their usefulness in cancer therapy [97] and suggesting the existence of different immunosuppressive mechanisms in cancer-bearing hosts. Notably, several reports have recently demonstrated that combined DGKα inhibition/silencing and PD-1/PD-L1 blockade synergistically enhance anticancer immunity (Figure 3). For example, Fu et al. recently reported that DGKα inhibition using R59022, R59949 and ritanserin enhanced the efficacy of anti-PD-1 therapy [98]. Moreover, Arranz-Nicolas et al. also showed the involvement of DGKα in CD3/CD28 and PD-1-mediated signal transduction pathways and the ability of DGKα inhibitors (R59949 and ritanserin) to cooperatively enhance immune checkpoint-targeted therapies in human T lymphocytes [99,100]. Furthermore, Okada et al. demonstrated that DGKα inhibition using DGKAI (see Section 5.1) and PD-L1 blockade synergistically suppressed the growth of HCC in vivo in an immune activity-dependent manner [90]. Therefore, the synergistic effect of DGKα inhibition and PD-1/PD-L1 blockade provides a promising strategy to strengthen the efficacy of immunotherapy in the treatment of cancer (Figure 3). The combination therapies with DGKα-specific inhibitors and anti-PD-1/PD-L1 antibodies, if established, will provide survival benefits for much greater numbers of advanced cancer patients.

### 5.3. Synergistic Effects of DGKα- and DGKζ-Inhibitions

In addition to DGKα, DGKζ reportedly restricts the intensity of T cell receptor signaling by metabolizing DG [12,76,78,80]. Moreover, DGKζ enhances the proliferation of human embryonic kidney 293 cells [101]. Therefore, we hypothesized that attenuation of DGKζ synergistically augments the enhancing effects of DGKα inhibition on cancer cell apoptosis and T cell function. Indeed, Takao et al. recently demonstrated that combined inhibition/silencing of DGKα and DGKζ synergistically provokes apoptosis of melanoma cells and interleukin-2 generation in T cells [102]. Therefore, a compound that inhibits both DGKα and DGKζ may be a promising anticancer treatment. Notably, Abdel-Magid reported that naphthyridineone compounds inhibit both DGKα and DGKζ and are useful as T cell function activators [103].

## 6. Conclusions

DGKα acts as an antiapoptosis/proproliferation factor in cancer cells. In contrast, DGKα attenuates the functions of T cells. Therefore, DGKα-selective inhibitors are expected to be ideal anticancer medicines because the inhibition of DGKα suppresses cancer cell proliferation and simultaneously activates T cell function (Figure 3). Moreover, synergistic effects of DGKα inhibition and PD-1/PD-L1 blockade would provide a promising new strategy for refractory cancer therapy (Figure 3). Furthermore, it is possible that the inhibition of DGKζ synergistically enhances the effects of DGKα inhibition (Figure 3). Immediate development of genuine DGKα-specific inhibitors (and DGKζ-specific inhibitors) is needed.
